# Papillary Thyroid Carcinoma Infiltrating a Parathyroid Adenoma: A Case Report

**DOI:** 10.7759/cureus.80961

**Published:** 2025-03-21

**Authors:** Adam McLuckie, Sydney B Thornton, Robin Andree, Scott O'Neil

**Affiliations:** 1 Medical School, Edward Via College of Osteopathic Medicine, Blacksburg, USA; 2 Pathology, Sovah Health - Martinsville Hospital, Martinsville, USA; 3 General Surgery, Sovah Health - Martinsville Hospital, Martinsville, USA

**Keywords:** papillary thyroid carcinoma, parathyroidectomy, parathyroid gland adenoma, primary hyperparathyroidism, subtotal thyroidectomy

## Abstract

Primary hyperparathyroidism due to parathyroid adenoma with concurrent papillary thyroid carcinoma is rare. Less frequently described is the histological interplay between these pathologies. This report describes the case of a 70-year-old female patient who presented with symptomatic hypercalcemia. Subsequent imaging was indicative of a left inferior lobe parathyroid adenoma and highly suspicious for left inferior lobe thyroid carcinoma. A subtotal thyroidectomy with isolated parathyroidectomy was performed. Postoperatively, the patient demonstrated normalization of calcium levels, parathyroid hormone, and resolution of symptoms. Histological evaluation demonstrated direct parathyroid adenoma invasion by the papillary thyroid carcinoma. This report demonstrates that although rare, parathyroid adenoma and papillary thyroid carcinoma can coexist and have the potential to complicate management.

## Introduction

Primary hyperparathyroidism (PHPT) is a condition characterized by elevated parathyroid hormone (PTH) levels and hypercalcemia due to excessive PTH secretion by one or more parathyroid glands. PHPT is often discovered due to elevated blood calcium levels on routine examination and/or symptoms of hypercalcemia such as polyuria, bone pain, muscle weakness, lethargy, constipation, and other digestive issues. Parathyroid adenoma (PTA) is the most common cause of PHPT and accounts for 80-85% of the cases of PHPT [1]. The primary management for symptomatic PHPT or asymptomatic PHPT in patients under the age of 50, an adjusted calcium level of 1 mg/dL above the upper limit of normal, or the presence of kidney or bone involvement, is parathyroidectomy. However, medical management, such as bisphosphonates, cinacalcet, or selective estrogen receptor modulators, may be considered for symptomatic control in a patient who is not a surgical candidate or refuses surgery with informed consent [2]. 

The 2022 WHO classification system differentiates thyroid neoplasms into three broad categories: benign lesions, low-risk neoplasms, and malignant thyroid neoplasms. Malignant thyroid neoplasms are further classified into follicular thyroid carcinoma, invasive encapsulated follicular variant papillary thyroid carcinoma, oncocytic carcinoma of the thyroid, papillary thyroid carcinoma (PTC), differentiated high-grade thyroid carcinoma, poorly differentiated thyroid carcinoma, and anaplastic thyroid carcinoma [3]. PTC is the most common type of thyroid cancer and accounts for 80% of thyroid carcinomas [4]. PTC may present asymptomatically, while symptoms such as a palpable neck mass, dysphonia, lymphadenopathy, and tenderness may be demonstrated. Surgery is the mainstay of treatment in PTC. Treatment options for PTC may include active surveillance, lobectomy, total thyroidectomy, total thyroidectomy with central neck dissection, and postoperative radioiodine therapy. Management of PTC depends on the tumor size, whether there is extrathyroidal extension, lymph node or distant metastasis, cytology, and a shared decision-making approach with the patient [5]. 

Though PHPT due to PTA and PTC are common endocrine pathologies separately [1,4] and a relationship has been previously described between these diseases [6,7], the co-occurrence of PTA and PTC is still considered rare [8,9]. Additionally, the histological interplay of these phenomena is extremely rare and often overlooked. While reports have been presented discussing the coexistence of PTA and PTC, evidence including the histologic extension of PTC into the PTA is limited and often addresses coexisting but distinct pathologies. In this case report, we present a patient with PHPT due to PTA, infiltrated by PTC (follicular variant). 

## Case presentation

**Figure 1:**
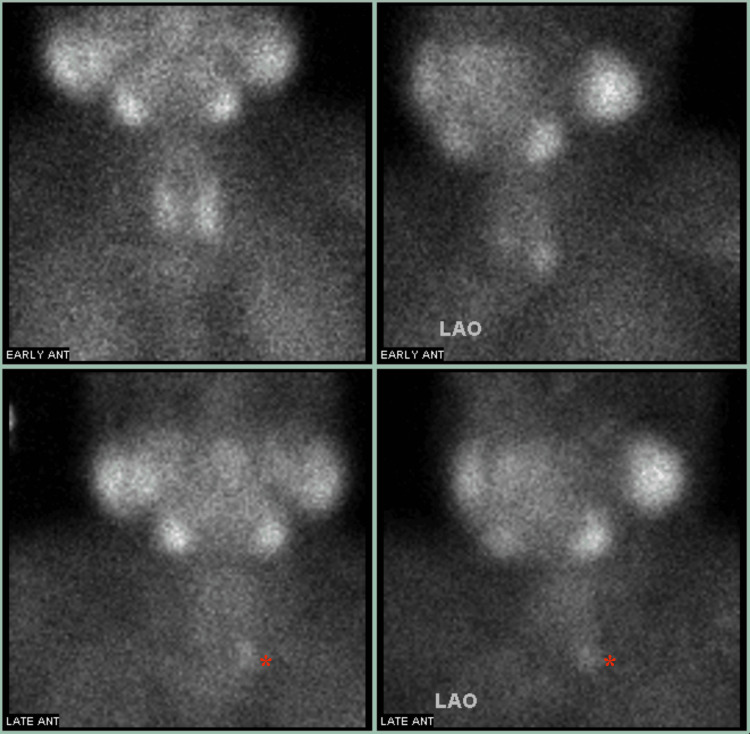
Tc-99m MIBI Scan of parathyroid gland demonstrating focused tracer retention of the left lower pole of the left lobe of the thyroid gland. The radiotracer was injected, and imaging was obtained in two different intervals: early and late (labeled). Initially, both the thyroid and parathyroid glands had radiotracer uptake due to the accumulation of Tc-99m in cells with high mitochondrial activity. Typically, during the late phase, the radiotracer would no longer be visible on imaging within the thyroid or parathyroid, considered the “washout” phase. However, due to the parathyroid adenoma containing hyperfunctioning tissue with high mitochondrial density, the PTA will uptake the Tc-99m focally for longer and therefore be visible on late imaging. The increased density can be seen in the lower left pole of the left lobe of the thyroid gland, labeled as a red asterisk (*). LAO: left anterior oblique; Tc-99m MIBI: technetium-99m sestamibi; PTA: parathyroid adenoma

**Figure 2:**
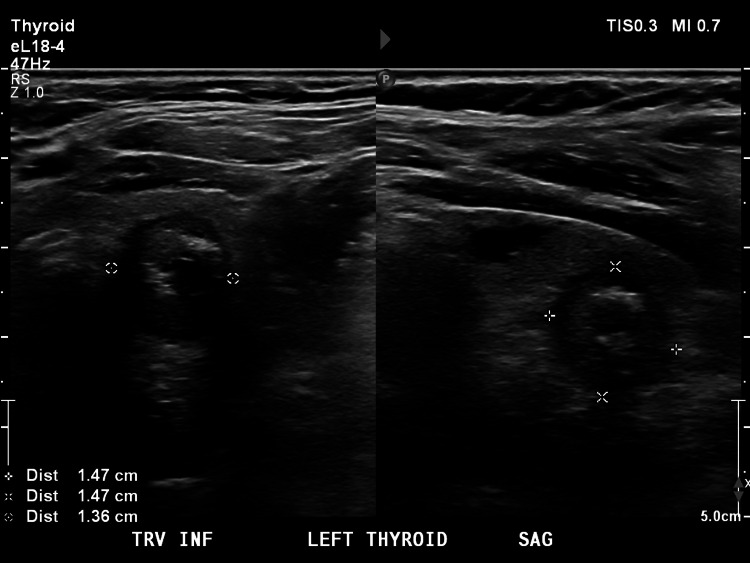
Ultrasound of thyroid gland demonstrating two nodules of the left inferior thyroid pole. ACR TI-RADS is a systematic approach to identifying if a thyroid nodule is suspicious for malignancy. It accounts for composition, echogenicity, shape, margin, and echogenic foci to determine the suspicion. The 1.47cm x 1.47cm x 1.36cm nodule labeled in the figure is considered highly suspicious (ACR > 7) according to the TI-RADS criteria due to the solid composition, hypoechoic nature, and echogenic foci. TRV: transverse; INF: inferior; SAG: sagittal; ACR: American College of Radiology; TI-RADs: Thyroid Imaging Reporting and Data System

A 70-year-old female patient presented with worsening confusion, sleeplessness, and diffuse musculoskeletal discomfort. Her past medical history included diabetes, hypertension, depression, anxiety, and chronic obstructive pulmonary disease. She did not have any prior neck radiation exposure. She was found to have PHPT with hypercalcemia and an elevated serum PTH (Table 1). Her serum thyroid-stimulating hormone (TSH) and thyroxine (T4) were both within normal limits (Table 1). Technetium-99m sestamibi (Tc-99m MIBI) scan was concerning for PTA of the left lower thyroid lobe (Figure 1). A thyroid ultrasound was obtained and demonstrated two nodules in the left inferior pole. One nodule was highly suspicious, and the second was a smaller, mildly suspicious nodule (Figure 2). Fine needle aspiration (FNA) was not pursued, as surgical intervention was warranted and requested. 

**Table 1:** Preoperative and postoperative laboratory results. Laboratory results showing preoperative hypercalcemia and primary hyperparathyroidism attributed to the papillary thyroid carcinoma and parathyroid adenoma. Postoperative results show resolution of both hypercalcemia and hyperparathyroidism.

Laboratory Test	Preoperative Result	Postoperative Result	Reference Values
Calcium	10.8 mg/dl	8.6 mg/dl	8.7-10.5 mg/dl
Parathyroid Hormone (PTH)	129 pg/dl	9 pg/dl	12-88 pg/dl
Thyroid Stimulating Hormone (TSH)	2.37 uIU/mL	0.847 uIU/mL	0.34-5.60 uIU/mL
Thyroxine (T4)	8.7 ug/dL	1.55 ug/dL	4.5-12 ug/dL

Intraoperatively, a mass was present on the left thyroid lobe and presented with extrathyroidal extension, anchoring the thyroid gland to the trachea and encompassing the left recurrent laryngeal nerve. A frozen biopsy was obtained, which indicated a follicular variant of papillary thyroid carcinoma. The left lower parathyroid gland could not be identified. Following the identification of the remaining three parathyroid glands, the tracheoesophageal groove and thyrothymic recess were explored. Without successfully identifying a PTA or the left inferior parathyroid gland, a subtotal thyroidectomy was performed. Since the recurrent laryngeal nerve was anchored to the base of the mass, it was carefully dissected around for nerve preservation. 

**Figure 3:**
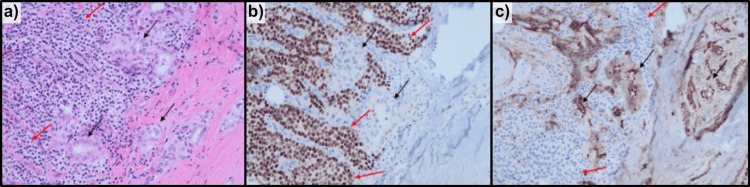
Transitional area between PTC (black arrows) and PTA (red arrows); PTC cells are infiltrating the PTA without clear definition between the two. (a) Hematoxylin and Eosin stain, (b) GATA-binding protein 3 stain, and (c) Thyroglobulin stain. (b) Positive GATA3 stain is suggestive of parathyroid origin. This is helpful to distinguish PTA from thyroid tissue; (c) Thyroglobulin stain is a specific marker for thyroid follicular cells and can confirm thyroid origin. PTC: papillary thyroid carcinoma; PTA: parathyroid adenoma

Postoperatively, the patient's repeat calcium and PTH were reduced to below-normal limits (Table 1). Additionally, she experienced marked symptomatic improvement, indicating the PTA appeared to have been resected. Further pathological evaluation indicated the PTA was completely surrounded and focally invaded by a follicular variant of PTC (Figure 3). Postoperatively, the patient received radioactive iodine 131 (RAI-131) ablative treatment to destroy any remaining thyroid tissue, and supplementation of calcium and levothyroxine to therapeutic levels. 

## Discussion

PHPT and thyroid carcinoma can be seen coexisting with multiple endocrine neoplasia (MEN) syndromes. However, patients with MEN, specifically type 2, are predisposed to the development of medullary thyroid carcinoma [10]. Excluding MEN syndromes, co-occurrence of PHPT and thyroid disease appears to have a female predominance and is reported to range from 2.8% to 47.1%. This is often due to the identification of benign nodules and microadenomas [7]. Excluding these typically benign findings, the incidence of PHPT and PTC has been described as ranging from 0.2% to 13.6% [7,8]. 

A well-known risk factor for the development of concurrent disease is prior neck radiation. Additionally, it is theorized that hypercalcemia may have an oncological effect on thyroid tissue, though evidence to support this is lacking [6,8]. However, lower preoperative PTH and calcium levels have been associated with a higher incidence of PTC and asymptomatic patients have been described with more aggressive carcinoma, which further challenges this theory. Patients with concurrent disease have also demonstrated higher rates of carcinoma capsular invasion [7]. True pathogenesis remains unknown and further studies are needed to adequately address this. 

Through histological evaluation of PTC, parathyroid infiltration has been observed ranging from 0.4% to 3.94% [11,12]. However, this is thought to be underdiagnosed due to the general surgical preference of preserving the parathyroid glands, limiting histological evaluation [1]. Previous histologic studies describe parathyroid infiltrative patterns as either direct invasion, invasion surrounded by pseudocapsule, or metastatic deposition [13]. Though parathyroid infiltration can occur, large-scale prospective evaluation of patients with PTC showed no difference in mortality when comparing PTC with minimal extrathyroidal extension to PTC with parathyroid extension without surrounding soft tissue invasion [14]. Adenoma invasion is likely underdiagnosed due to parathyroid gland preservation, necessitating further investigation into its true incidence, invasion characteristics, prognostic implications, and management, as current data is limited. 

Preoperative evaluation of PHPT through ultrasound and Tc-99m MIBI scan are standard practices, while PTC is generally evaluated through ultrasound and FNA [4,7]. Through increased use of ultrasound, PTC prevalence has increased [4], which may lead to an increased rate of identifying concurrent pathology. However, in evaluating patients with suspected concurrent pathology, using multiphasic multidetector four-dimensional computer tomography (4D-CT) may offer benefits over traditional imaging modalities to differentiate between PTA, thyroid nodules, and PTC. Specifically, contrast-enhanced 4D-CT may be beneficial in differentiating intrathyroid PTAs from colloid nodules and PTC [15]. However, a combined 4D-CT and Tc-99m MIBI scan imaging approach may provide marginally better diagnostic accuracy, localization of PTAs, and preoperative detailed anatomic information for surgery [16]. 

## Conclusions

This case offers a unique example of PTA and PTC co-occurrence and histologic interplay. Given the high prevalence of PTA, PTC expansion and extension into the adenoma is a rare finding that may go underdiagnosed and has the potential to complicate a typically routine procedure. A high clinical index of suspicion should be maintained when evaluating and treating patients with suspected dual pathology. A detailed evaluation of history, physical examination, laboratory values, imaging, and gross anatomy inspection during surgery should be used to consider concomitant PTA and PTC. This case highlights the importance of thorough preoperative imaging in conjunction with postoperative histologic and laboratory evaluation, as well as remaining vigilant to the possibility of overlapping pathologies. Though associations have been seen through case reports, retrospective, and observational studies, guidelines in evaluation and management are lacking in addition to trials evaluating prognosis with concurrent pathology. By presenting this report, we hope to raise awareness of the evaluation and management of the coexistance and histological interplay of PTA and PTC. 
